# Measuring Psychological Change during Cognitive Behaviour Therapy in Primary Care: A Polish Study Using ‘PSYCHLOPS’ (Psychological Outcome Profiles)

**DOI:** 10.1371/journal.pone.0027378

**Published:** 2011-12-15

**Authors:** Slawomir Czachowski, Paul Seed, Peter Schofield, Mark Ashworth

**Affiliations:** 1 Collegium Medicum, Nicolaus Copernicus University, Torun, Poland; 2 Department of Primary Care and Public Health Sciences, King's College London, London, United Kingdom; RAND Corporation, United States of America

## Abstract

**Background:**

Psychological outcome measures are evolving into measures that depict progress over time. Interval measurement during therapy has not previously been reported for a patient-generated measure in primary care. We aimed to determine the sensitivity to change throughout therapy, using ‘PSYCHLOPS’ (Psychological Outcome Profiles), and to determine if new problems appearing during therapy diminish overall improvement.

**Methods:**

Responses to PSYCHLOPS, pre-, during- and post-therapy were compared. Setting: patients offered brief cognitive behaviour therapy in primary care in Poland.

**Results:**

238 patients completed the pre-therapy questionnaire, 194 (81.5%) the during-therapy questionnaire and 142 the post-therapy questionnaire (59.7%). For those completing all three questionnaires (n = 135), improvement in total scores produced an overall Effect Size of 3.1 (2.7 to 3.4). We estimated change using three methods for dealing with missing values. Single and multiple imputation did not significantly change the Effect Size; ‘Last Value Carried Forward’, the most conservative method, produced an overall Effect Size of 2.3 (1.9 to 2.6). New problems during therapy were reported by 81 patients (60.0%): new problem and original problem scores were of similar magnitude and change scores were not significantly different when compared to patients who did not report new problems.

**Conclusion:**

A large proportion of outcome data is lost when outcome measures depend upon completed end of therapy questionnaires. The use of a during-therapy measure increases data capture. Missing data still produce difficulties in interpreting overall effect sizes for change. We found no evidence that new problems appearing during therapy hampered overall recovery.

## Introduction

Psychological outcome measures are evolving into measures that no longer report end-of-therapy outcomes alone, but also report progress over time. There are two reasons for this development. Firstly, repeated measures generate a longitudinal record of psychological state allowing both patient and therapist to track progress over time [1]. Secondly, data capture at multiple time points offers an alternative to the shortcoming of instruments which can only measure change once therapy and a post-therapy questionnaire have both been completed. First session/last session methods are inherently problematical because change scores cannot be derived for patients failing to complete therapy. By capturing scores during the course of therapy, change scores can be derived up until the point where the patient no longer participates in therapy or questionnaire completion.

### ‘PSYCHLOPS’ as a patient-generated outcome measure

We have previously validated ‘PSYCHLOPS’ (‘Psychological Outcome Profiles’), a patient-generated (“idiographic”) psychological outcome measure for use in primary care [2], [3]. However, it has been specifically designed as a first session/last session measure hence its usefulness has been restricted by high attrition rates which are common during talking therapies. In two validation studies involving PSYCHLOPS, firstly comparing it with CORE-OM and secondly with HADS, completion rates were 47% and 34%, respectively [2], [3]. These studies may have exaggerated data loss, since patients were excluded if they failed to complete either PSYCHLOPS or the comparator instrument. Nevertheless, in routine use, it is not atypical for only a third of patients can be expected to complete both a pre- and post-therapy outcome measure [4]. Findings based solely on measures obtained on completion of therapy may be subject to bias and are likely to provide a more optimistic assessment of outcomes since those dropping out of therapy are more likely to do so because of non-responsiveness than because of rapid recovery [5].

PSYCHLOPS addresses three domains: Problems (two questions, P1 and P2), Function (one question, F1) and Wellbeing (one question, W1). In the pre-therapy version, patients are asked to describe their main Problem (in a freetext box) and to score it; the process is repeated for the Function question but the Wellbeing question is a response to a nomothetic scale (without an idiographic component to this domain). The post-therapy version requires the therapist to transcribe the original freetext Problem and Function responses – patients are then asked to re-score these items and also to score Wellbeing. Although not used for the outcome score, PSYCHLOPS also contains client and therapist validation questions, asking both for an assessment of change since the beginning of therapy, with responses coded on a nomothetic scale.

### Using PSYCHLOPS for repeated measures

We have adapted PSYCHLOPS (version 5) so that it now incorporates a during-therapy version allowing repeated administration and a repeated measures analysis. The revised pre-therapy version of PSYCHLOPS has only required small modifications to the introduction and layout. The new during-therapy version follows a similar format but we have introduced an additional question asking whether any new Problems have emerged during therapy and if so, asking the patient to describe the most troubling new problem (P3) in a freetext box and to score it. Finally, the revised post-therapy questionnaire has been modified to mirror the additional question in the during-therapy version by asking the client to score problems which arose during therapy, now that therapy has been completed.

Introducing this during-therapy version of PSYCHLOPS enlarged the ‘family’ of PSYCHLOPS questionnaires and increased the likelihood that change data will be collected even if the patient terminates therapy early. However, it raises questions about the meaning of the data and about the best ways to analyse such repeated measures data given the idiographic design. We also needed to test its psychometric properties particularly to explore six change parameters:

- Sensitivity to change, i.e. the effect size of change, measured using both the during-therapy and end-of-therapy PSYCHLOPS.
- The interpretation of missing change scores as a result of therapy and questionnaire non-completion, and the use of various imputation methods to overcome missing scores.
- Whether the appearance of new problems during therapy would partially offset any apparent improvement in the original pre-therapy scores.
- Internal reliability of the problem scores in the new during and end of therapy PSYCHLOPS instruments.
- Whether change appears broadly linear or curvilinear.
- The validity against participant and therapist rated change.

We therefore devised a study to answer these questions.

## Methods

### Study design

We conducted a longitudinal survey. Patients were asked to complete PSYCHLOPS before starting cognitive behaviour therapy therapy (immediately prior to commencement of the first therapy session), at least once during therapy (at the end of the second and of subsequent sessions) and following completion of therapy (at the end of the final session).

### Setting and practitioners

We conducted our study in a routine primary care setting in Poland. A total of 35 general practitioners (GPs) were recruited, all singlehanded and linked through a postgraduate network to Copernicus University, Torun. Their practices were predominantly located in urban areas, although seven were in a rural setting.

As is usual in Poland, talking therapy was provided by the GPs themselves and was brief, consisting of three or four, 30 minute sessions. Talking therapy followed the principles of cognitive behavioural therapy (CBT) and each participating GP had received postgraduate training in applying brief CBT to routine practice. Psychologists also provide talking therapy in Poland, but usually within a secondary care context as part of the psychiatric team. Since we wanted to study the response to CBT in primary care, psychologists were not included in the study.

### Patients

Those eligible for the study were all patients attending participating GPs who were offered and accepted referral for brief CBT during the 6-month study period. Routine practice determined the age range of participants: female patients were aged 18–60 years and males aged 18–65 years. Patients who were outside these age ranges were referred to other services. Brief CBT was offered to those with psycho-somatic symptoms, anxiety or depression. Patients were excluded if they had a current history of psychotic illness, substance abuse, an organic illness impairing mental function or were insufficiently literate.

### Ethical considerations

The therapeutic intervention (brief CBT) was not modified for study participants. However, completion of outcome measures was not routine practice and this had to be incorporated into the therapy protocol. Ethical permission was granted by the Ethics Committee, Kuyavian-Pomeranian Doctors Chamber, University of Torun, code: OIL-67/KB/589/2008 (October 2008).

### Statistical methods

We constructed a longitudinal dataset consisting of all questionnaire responses to PSYCHLOPS, pre-, during- and post-therapy. The scoring system for PSYCHLOPS allocates a score of zero to five points to each question. There are four questions in all, producing an overall PYSCHLOPS score ranging from zero to 20 (where someone only offers one problem the rating for that problem is doubled). The additional question asking about new problems arising during therapy is contained in the during- and post-therapy questionnaires and is also scored from zero to five but not included in a new composite score to keep consistency with traditional first-session/last-session usage. Data from the two validation questions in the post-therapy questionnaire were collected: a nomothetic question asking the patient how they felt post-therapy compared to pre-therapy, and a similar question asking the therapist to score recovery (both scored from zero to five).

#### Sensitivity to change: effect size

The design of the study was exploratory rather than hypothesis testing [6], [7], [8]. Sensitivity to change (‘responsiveness’) was explored by calculating Cohen's ‘effect size’ and calculated as the change score divided by the pre-therapy standard deviation (SD) [9]. Values derived for the effect size represent the number of SDs by which the initial score has changed after therapy and a value of 0.8 or greater is generally considered large for health service related outcomes [10]. Parameters are reported with their 95% Confidence Intervals (CIs).

#### The interpretation of missing change scores

We explored the five different ways of calculating change on the effect size [11], [12]. Firstly, the effect size was calculated for all patients completing all three pre-, during- and post-therapy questionnaires. This method is termed ‘listwise deletion’ or ‘complete case analysis’ since all cases with missing values are deleted from the analysis. Secondly, the effect size was calculated for all those entering the study with no attempt made to replace missing data when patients failed to complete subsequent during- and post-therapy questionnaires. This method is termed ‘pairwise deletion’ (or ‘available case analysis’) and is based on analysis employing all available data. Thus, in this instance, an effect size is calculated for everyone completing pre- and during-therapy, during- and post-therapy and also pre- and post-therapy questionnaires. Although overlapping, these three datasets may include different cases.

Both pairwise deletion and listwise deletion are valid provided the data is “Missing Completely at Random” (MCAR) [13], i.e. the subjects with missing values do not differ systematically in any important respect from those with complete data in terms of measurable or un-measurable criteria. However, in a trial of talking therapy, it is always likely that those who drop out may differ in some way from subjects who continue.

Imputation methods, which use the known differences between subjects that drop out and those which do not, rely on the less restrictive “Missing At Random” (MAR) assumption – that systematic differences between the unknown and known values may occur, but are matched by differences in the known values used as predictors, and that the bias caused by the systematic differences can therefore be removed by appropriate methods. Both single imputation and multiple imputation were used.

Thirdly, we used single imputation: imputed values for the interim and final scores were calculated where the true values was not measured, using best-subset linear regression [14]. For the interim scores, baseline values of age, gender and the three PSYCHLOPS subscales were used as predictors. For the final scores, the interim values of the subscales were also used. A fixed practice effect was included in the imputation model and variance of the estimates was adjusted for clustering by practice using the Huber-White method. The best-subset method ensured that values were imputed appropriately even when not all predictors were known. Single imputation treats plausible values as if they are additional data which in turn tends to give narrower standard errors and more significant p values than are justified by the data.

Fourthly, we used Multiple Imputation in which not just one value is substituted but a series of ‘stochastic’, or random values are imputed based on the predicted value and error of estimation. These stochastic values introduce estimation error variance into the imputed data and give valid standard errors and significance tests (if the MAR assumption is valid). Although 3–5 imputations are generally considered sufficient, we sought to maximise the benefit of this approach by using 20 imputations. Multiple imputation therefore includes corrections for potential bias as well as giving a valid standard error; it may be regarded as the most reliable and least subject to bias of the methods considered here. We incorporated the same predictor variables as used in the single imputation model, for both interim and final scores.

Fifthly and finally, we used the technique of ‘last value carried forward’ (LVCF). The last recorded score of each patient was used to create a dummy score for subsequent questionnaires, thus assuming no further improvement. The effect size was recalculated based on the entire initial sample, with missing values replaced by these unchanged dummy scores. This method is widely used, but it is known to underestimate changes over time, and again, to give ‘anti-conservative’ standard errors and significance tests. For all five effect size scores, the standard errors were adjusted for the effect of clustering by clinical practice.

#### Incorporating scores for new problems arising during therapy

We conducted separate analyses of effect sizes for the original Problem (P1) and for the new Problem (P3). However, throughout the analysis, total PSYCHLOPS scores excluded any weighting for P3, if reported.

#### Internal reliability testing

Internal reliability was tested by calculating Cronbach's alpha for the three domain scores in PSYCHLOPS. A score of 0.70 is generally considered as demonstrating satisfactory internal consistency [15].

#### Tests of linearity

In order to test for non-linearity, two generalised linear models (GLM) were fitted to the original dataset using maximum likelihood estimations, one with linear and one with categorical effects for time. The likelihood ratio test was used to determine the significance of any difference between the two models and therefore whether there is any evidence of non-linearity. This method was repeated in the multiple imputation dataset but with one difference. Multiple imputation methods do not permit likelihood ratios and therefore the significance of any deviation from linearity has to be estimated using a categorical effects model.

#### Concurrent and convergent validity testing

A subsidiary validation exercise was conducted. Firstly, the correlation was determined between overall PSYCHLOPS change scores and self reported recovery as recorded by the post-therapy validation question included in the standard PSYCHLOPS questionnaire (concurrent validity). This question states: “Compared to when you started therapy, how do you feel now?”. Secondly, the correlation was determined between self reported recovery and therapist reported recovery (the validation question completed by the therapist), also included in the standard questionnaire (convergent validity). This question states: “Now that therapy has finished, how would you describe the client overall?”. Both correlations were based on 6-point ordinal scales and were analysed using the non-parametric test, Spearman's *rho*. Values between 0.3 and 0.7 generally denote a moderate correlation.

Results were analysed using STATA version 8.2.

## Results

### Patient sample

All patients accepting the offer of CBT during the 6-month study period completed the initial PSYCHLOPS questionnaire. A total of 243 patients entered the study but five first session questionnaires were unusable leaving a total of 238 patients with analysable pre-therapy data. The mean age of this sample of 238 was 41.5 years (range 18–64) and 78.1% were female.

During-therapy questionnaires were completed by 194 (81.5%) of the original sample of 238. Just seven completed more than one during-therapy questionnaire; because of small numbers, these were ignored and only the results of the first during-therapy questionnaire were included in the study. Non-completion of a during-therapy measure was apparently not random: those who failed to complete the during-therapy questionnaire were more likely to be female (21.0% of females compared to 7.8% males, Pearson χ^2^ = 4.64; p = 0.04), though neither age, nor initial PSYCHLOPS scores were statistically related to completion of a during-therapy measure.

Post-therapy questionnaires were completed by 142 (59.7%) patients of whom 135 (56.7%) had completed all three questionnaires. These patients constituted a longitudinal cohort for whom data were available over three time points. There were no significant differences in age nor gender between completers and non-completers of the post-therapy questionnaires. Post-therapy completers had higher pre-therapy scores than the 103 non-completers: 11.9 compared to 11.1, t = −2.59, p = 0.01, and higher during-therapy scores than non-completers: 11.3 compared to 9.9, t = −2.48, p = 0.01.

### Therapy sessions and practitioners

All patients received CBT and the number of therapy sessions was very similar throughout the sample: mean, 3.1; range, 3–5; SD, 0.28. The mean number of patients recruited by each practitioner was 5.4, range 1–10.

### PSYCHLOPS scores

Mean PSYCHLOPS scores demonstrated improvement over the course of therapy. The mean overall change for those completing all three questionnaires based on each of the selected methods for dealing with missing data are presented in Table 1.

**Table 1:** PSYCHLOPS scores: mean scores.

	‘Completers’*(N = 135)*	‘Starters’ *(N = 238, 194, 142, respectively)*	Single imputation *(N = 238)*	Multiple imputation *(N = 238)*	LVCF* *(N = 238)*
Pre-therapy	15.8	15.5	15.5	15.5	15.5
During-therapy	11.3	10.9	10.8	10.8	11.7
Post-therapy	6.4	6.3	6.1	6.3	8.5
**Overall change**	**9.4 (8.3 to 10.4)**	**9.5 (8.5, 10.5)**	**9.3 (8.6, 10.0)**	**9.5 (8.5, 10.5)**	**6.9 (5.8, 8.1)**

*‘LVCF’ = Last Value Carried Forward.

Figures are means and 95% confidence intervals (CIs) in brackets.

PSYCHLOPS scores (ignoring any new problems) increased in just one out of 142 (0.07%) patients completing therapy. A further 2 (1.4%) showed no change. A larger number showed unchanged during-therapy scores: 11 (5.7%), of whom 3 failed to complete; a further 9 of 194 (4.6%) showed elevated during-therapy scores of whom 2 failed to complete their therapy.

### PSYCHLOPS sensitivity to change

PSYCHLOPS change scores were converted into effect sizes for both cohorts of patients and the results are presented in Table 2. The overall effect sizes for ‘completers’ and ‘starters’ were 3.1 (2.7 to 3.4) and 3.1 (2.8 to 3.4), respectively. Similar effect sizes were obtained using single and multiple imputation: 3.1 (2.8 to 3.3) and 3.2 (2.9 to 3.5), respectively, whereas LVCF produced a smaller effect size of 2.3 (1.9 to 2.6).

**Table 2:** PSYCHLOPS sensitivity to change: the Effect Size.

	‘Completers’*(N = 135)*	‘Starters’ *(N = 194, 142, respectively)*	Single imputation *(N = 238)*	Multiple imputation *(N = 238)*	LVCF* *(N = 238)*
Pre-therapy to During-therapy	1.5	1.5	1.5	1.6	1.2
**Overall change**	**3.1 (2.7, 3.4)**	**3.1 (2.8, 3.4)**	**3.1 (2.8, 3.3)**	**3.2 (2.9, 3.5)**	**2.3 (1.9, 2.6)**

*‘LVCF’ = Last Value Carried Forward.

Figures are means and 95% confidence intervals (CIs).

We also compared effect sizes for each of the domains within PSYCHLOPS. The overall effect sizes for the smaller cohort of ‘completers’ were: Problem domain: 2.8 (2.6 to 3.0); Function domain: 2.2 (2.0 to 2.5); Well-being domain 2.5 (2.3 to 2.8). For the larger cohort of ‘starters’ the effect sizes were: 2.1 (1.9 to 2.3); 1.7 (1.5 to 1.9); 1.7 (1.5 to 1.9), respectively. In both these analyses, the Problem domain effect size was larger than that of the other domains: paired t = 2.47; p = 0.015 (‘completers’) and paired t = 4.71; p<0.001 (‘starters’).

### PSYCHLOPS scores for new problems arising during therapy

When asked post-therapy, 100 (74.1%) of the 135 ‘completers’ reported that new problems (P3) had arisen during therapy. Only 86 of these 100 patients reported new problems on their first during-therapy questionnaire. In other words, some patients reached the end of therapy and declared that new problems had occurred during therapy although they did not record these new problems on their during-therapy questionnaire. Conversely, some reported the emergence of new problems on their during-therapy questionnaire but these were not declared on the post-therapy questionnaire.

A full dataset with scores for new problems both during and after therapy was available for 81 patients. We compared the changes in the new problem, P3, (first declared on the during-therapy questionnaire) and the original problem, P1 (declared on the pre-therapy questionnaire). For this cohort, the mean during-therapy score for P3 was 3.3 (3.0 to 3.6), falling to 1.8 (1.5 to 2.1) post-therapy, and the mean score for P1 was 4.22 (4.0 to 4.4) pre-therapy, falling to 3.0 (2.8 to 3.2) during therapy and falling further to 1.6 (1.3 to 1.9) post-therapy.

The effect sizes which mirror these changes in mean scores were also calculated for the Problem domain, representing the change (improvement) in the new problem between the during- and post-therapy questionnaires: the effect size was 1.13 (0.90 to 1.37) for P3 and 1.58 (1.33 to 1.82) for P1, over the same time period.

We compared the effect sizes for those reporting a new problem on their during-therapy questionnaire with those who did not report any new problems, in order to determine if overall recovery (pre-therapy to post-therapy) differed between the two groups. The mean effect sizes were 2.94 (2.64 to 3.23) and 2.94 (2.55 to 3.34) for those with and without new problems, respectively, t = 0.24; P = 0.98.

Therapy drop-out rates were compared between those who declared a new problem during-therapy and those who did not. This cohort consisted of all 194 patients who completed during-therapy questionnaires, of whom 135 (69.6%) declared a during-therapy problem. Drop-out rates were lower among those declaring a new problem (14/100, 14.0%) compared to those who did not declare a new problem (45/94, 47.9%), χ^2^<0.001.

### Internal reliability

Values for Cronbach's alpha were calculated for the four component questions of PSYCHLOPS at each of the three time points of the study. For all those completing the pre-therapy version, the alpha value was 0.81 (95% CI, 0.77 to 0.85); during-therapy the alpha was 0.85 (95% CI, 0.82 to 0.89); post-therapy the alpha was 0.88 (95% CI, 0.85 to 0.91). These values indicator satisfactory internal reliability.

### Linearity of change scores

Measurements were compared pre-therapy, mid-therapy and post-therapy (after the final session). The mean change in total PSYCHLOPS score after completion of therapy (−9.4; 95% CI's, −10.0 to −8.9) was almost exactly twice that at the mid-therapy time point (−4.6; 95% CI's, −1.07 to 0.73), P = 0.71. There is therefore no evidence of a deviation from linearity. These results are based on multiple imputation estimates (20 imputations) and random-effects maximum likelihood regression.

### PSYCHLOPS validation scores

Two calculations of validity were conducted. We found a significant correlation between overall change recorded on PSYCHLOPS and self reported change, Spearman's *rho*, 0.60 P<0.001. Similarly, we found a significant correlation between self and therapist reported recovery, Spearman's *rho*, 0.61, P<0.001.

## Discussion

### Main findings: during-therapy version of PSYCHLOPS

The introduction of a during-therapy PSYCHLOPS outcome measure has resulted in a number of advantages. Firstly, the during-therapy measure boosted the proportion of patients with valid change scores from 56.7% to 81.5%, because change data, derived from the during-therapy scores, were available even when patients did not complete a post-therapy measure. Secondly, PSYCHLOPS remains highly responsive to change and change can now be measured using PSYCHLOPS at intervals during therapy without having to wait until completion of therapy. The change during therapy was consistent with a linear model of change: using all five different methods to handle missing values, the change occurring up to the mid-point of therapy was almost exactly half the overall change for each method. Thirdly, the characteristics of patients dropping out of CBT mid-therapy can be determined using the during-therapy questionnaire – they were similar in age and gender to completers but had lower pre-therapy and during-therapy PSYCHLOPS scores.

The three domains of PSYCHLOPS followed a similar change pattern. The largest effect sizes were noted for the Problem domain. Although there was some overlap of confidence intervals, the effect size of the Problem domain was significantly larger than that of the other domains, implying that the Problem domain makes the dominant contribution to the high responsiveness to change of PSYCHLOPS.

The during-therapy version of PSYCHLOPS provided additional information on new problems arising during the course of therapy. We had been concerned that new problems might be less responsive to therapy and might overshadow the recovery in the original problem. Our findings, particularly the identical effect sizes for change at the end of therapy, suggest that the appearance of new problems during therapy did not hamper overall recovery. Moreover, the magnitude of new problems arising mid-therapy was significantly smaller than scores elicited at the outset for the original problems. Interestingly, patients who declared new problems during therapy were significantly more likely to complete therapy than those who did not.

### Interpretation of missing values: sensitivity analysis

Given that data attrition in studies of talking therapy can reduce the final dataset by two-thirds [4], we planned a rigorous analysis based on five different methods following best practice recommendations for handling missing data [11], [12]. There were no significant differences (based on overlapping confidence intervals) between analysis based on the sample of ‘completers’, the sample of ‘starters’, nor the analyses generated by two imputation techniques. We had postulated beforehand that confining our analysis to those who completed therapy might produce an over-optimistic assessment of effect size. This was not borne out by the results. The striking similarity between the effect sizes calculated in four ways provides some support for the robustness of these estimates. The high level of agreement between the imputed and non-imputed methods suggests that the Missing Completely At Random assumption is reasonable in this study and that methods based only on completed data may be reliable.

The fifth way of calculating effect size was based on ‘LVCF’. As such, it replaces missing values on the assumption that no further recovery nor deterioration will take place. In reality, this is likely to generate a highly cautious interpretation of the effect size since natural recovery of problems would be expected without any intervention and we had little evidence of deterioration in the sample who remained in the study (with the exception of just one case out of 142). As expected, the effect sizes based on this method of calculation were smaller. It is probably best to consider the value derived from LVCF of 2.26 (1.9, 2.6) as a cautious under-estimate of the effect size. The true mean effect size for the whole group is likely to lie closer to the value found by the other four estimates.

In summary and in alignment with the original aims, our study involving the use of the new during-therapy questionnaire has generated more data with lower data attenuation rates than in earlier studies confined to pre- and post-therapy analysis and has enabled multiple imputation methods to be used to generate putative missing values and reinforce interpretations of change scores.

### Validation of PSYCHLOPS

More formal psychometric testing confirmed satisfactory internal reliability, concurrent and convergent validity with values exceeding the minimum standards determined beforehand.

### Limitations of the present study

The setting in primary care in Poland tests the generalisability of our earlier UK findings but data should be extrapolated to other settings with caution. Nevertheless, our findings confirm the validity and reliability of PSYCHLOPS on formal testing in primary care outside the UK. In Poland, unlike in the UK, CBT in primary care is usually provided by GPs during extended 30-minute appointments. Courses of therapy averaging three sessions are considerably shorter than in the UK though is congruent in duration if not perhaps in theory with ultra-short therapies [16]. This short duration of therapy probably contributed to the high post-therapy questionnaire completion rate.

The use of outcome measures is not usual practice in Poland and both response rates and responses may have been biased by their novelty factor. Because of this unfamiliarity, and the experiences of piloting, we only included one outcome measure in our study. We did not have a comparator instrument to provide standardised outcome measurement although in a previous study, PSYCHLOPS scores showed correlation coefficients (Spearman's rho) with CORE-OM of 0.65 and 0.74, pre- and post-therapy respectively [2].

Selection bias may have contributed to our findings as almost four-fifths of respondents were female. This proportion was higher than that found in previous validation studies which were 71% [2] and 56% [3] although it is uncertain in which direction this may have influenced our findings.

### Further work

PSYCHLOPS' responsiveness to change has been tested in a Polish primary care setting and, given the diversity of European primary care, needs to be tested in further settings. Although the present study only analysed the quantitative data from PSYCHLOPS, the three freetext boxes provide a rich source of qualitative data. Further research is needed on the qualitative components of change and whether there is a relationship between the quantitative and qualitative data. For example, sub-types of patients may be identified whose problems respond in different ways to courses of therapy.

Further work is also needed to compare different approaches to the incorporation of the score for new problems (P3) arising during the course of therapy. One approach would be to continue to pro-rate the Problem questions but where there are scores for three Problems, to select only the two highest scoring questions. Alternatively, all three Problem scores could be pro-rated, again producing a maximum possible score of 10, therefore diminishing the maximum contribution of P1 to a value of 3.33 rather than its current value of 5.0. We hope to conduct a further study to compare the findings from several different methods of calculating the contribution of P3 values.

### Implications of findings

PSYCHLOPS has proved to be feasible for use in a non-UK primary care setting and its high sensitivity to change after talking therapy has been confirmed. It shifts the focus of evaluation of health service treatment away from professionally derived concepts towards issues of importance to patients. As such, it is concordant with the recent development of Patient Reported Outcome Measures (PROMS) which attempt to harness feedback from patients about outcomes through reliable and valid patient reported health instruments [17]. Although PROMS currently cover a range of physical health conditions, and are being piloted for use in long term conditions affecting physical health, there are currently no available PROMS for patients using mental health services (*ibid*). Given the feasibility of PSYCHLOPS in this and previous studies, a case could be made for developing and promoting patient reported health instruments (PROMS) for mental health service evaluation.

### Further details about PSYCHLOPS

Further details about the latest version (version 5), scoring method and how to obtain copies of PSYCHLOPS are available on the website: www.psychlops.org.uk.
